# Influence of ambient humidity on the attachment ability of ladybird beetles (*Coccinella septempunctata*)

**DOI:** 10.3762/bjnano.7.123

**Published:** 2016-09-22

**Authors:** Lars Heepe, Jonas O Wolff, Stanislav N Gorb

**Affiliations:** 1 Department of Functional Morphology and Biomechanics, Kiel University, Am Botanischen Garten 1–9, D-24118 Kiel, Germany; 2 Mads Clausen Institute, University of Southern Denmark, NanoSYD Alsion 2, 6400 Sønderborg, Denmark; 3 Department of Biological Sciences, Macquarie University, Sydney, NSW 2109, Australia

**Keywords:** adhesion, beetle, biomechanics, force measurement, friction, insect, locomotion, surface

## Abstract

Many insects possess adhesive foot pads, which enable them to scale smooth vertical surfaces. The function of these organs may be highly affected by environmental conditions. Ladybird beetles (Coccinellidae) possess dense tarsal soles of tenent setae, supplemented with an adhesive fluid. We studied the attachment ability of the seven-spotted ladybird beetle (*Coccinella septempunctata*) at different humidities by horizontal traction experiments. We found that both low (15%) and high (99%) relative humidities lead to a decrease of attachment ability. The significantly highest attachment forces were revealed at 60% humidity. This relationship was found both in female and male beetles, despite of a deviating structure of adhesive setae and a significant difference in forces between sexes. These findings demonstrate that not only dry adhesive setae are affected by ambient humidity, but also setae that stick due to the capillarity of an oily secretion.

## Introduction

Substrate attachment plays an important role in the niche occupation of plant-dwelling insects, since it is substantial for resting and locomotion in a complex environment. Consequently, a high diversity of friction and adhesion enhancing structures has evolved among insects [1–2]. Several studies showed that not only the intrinsic structure of an attachment organ determines its function, but also environmental parameters, such as the surface roughness or/and chemistry of the substrate [1,3–9]. Also the ambient temperature and humidity may affect the attachment ability of adhesive organs, as it was shown in the dry adhesive pads of geckoes [10–13] and spiders [14]. For small arthropods these conditions may highly vary micro-spatially, especially in the boundary layer of plant leaves [15]. Furthermore, on most surfaces there is an adsorbed film of water molecules, with a thickness and mechanical properties highly influenced by ambient humidity [16–17]. Due to its polarity it can work as a bonding agent between two surfaces, and therefore have a substantial effect on adhesion [18–21]. In insects the terminal contact elements of tenent setae are not dry, but rather wetted by a fluid secretion that is usually a micro-emulsion of water and oil, with varying fractions of substances [22–31]. The mixture of both polar and non-polar substances presumably helps to wet both hydrophobic and hydrophilic surfaces building a fluid meniscus between the seta and the substrate to yield high capillary forces (wet adhesion). The fluid can increase the contact area by filling minute cavities of micro- and nano-rough surfaces, where setae otherwise cannot adapt to, and prevents slipping of the foot due to its specific rheological properties [31–35]. It was demonstrated that the presence of these secretions is crucial for the function of insect adhesive organs [6,36]. Adhesion is affected, if the water content of the secretion is manipulated by a water adsorbing substrate [28]. However, it is not clear, how the thickness and composition of the secretion fluid film are affected by ambient humidity.

In the present study, force measurements with tethered seven-spotted ladybirds (*Coccinella septempunctata*) were carried out in an environmental chamber at various controlled humidities. Experiments were carried out with female and male beetles, which differ in the structure of their adhesive pads [6]. Males possess tenent setae with discoidal tips that are assumed to be an adaptation to securely attach to the smooth elytrae of the female during both copulation and mate guarding [6,37]. Two main questions were asked. (1) Does ambient humidity influence the attachment ability of the wet adhesive system of *C. septempunctata*? (2) Is the attachment ability in both sexes likewise affected by ambient humidity?

## Experimental

### Animals

**Figure 1:**
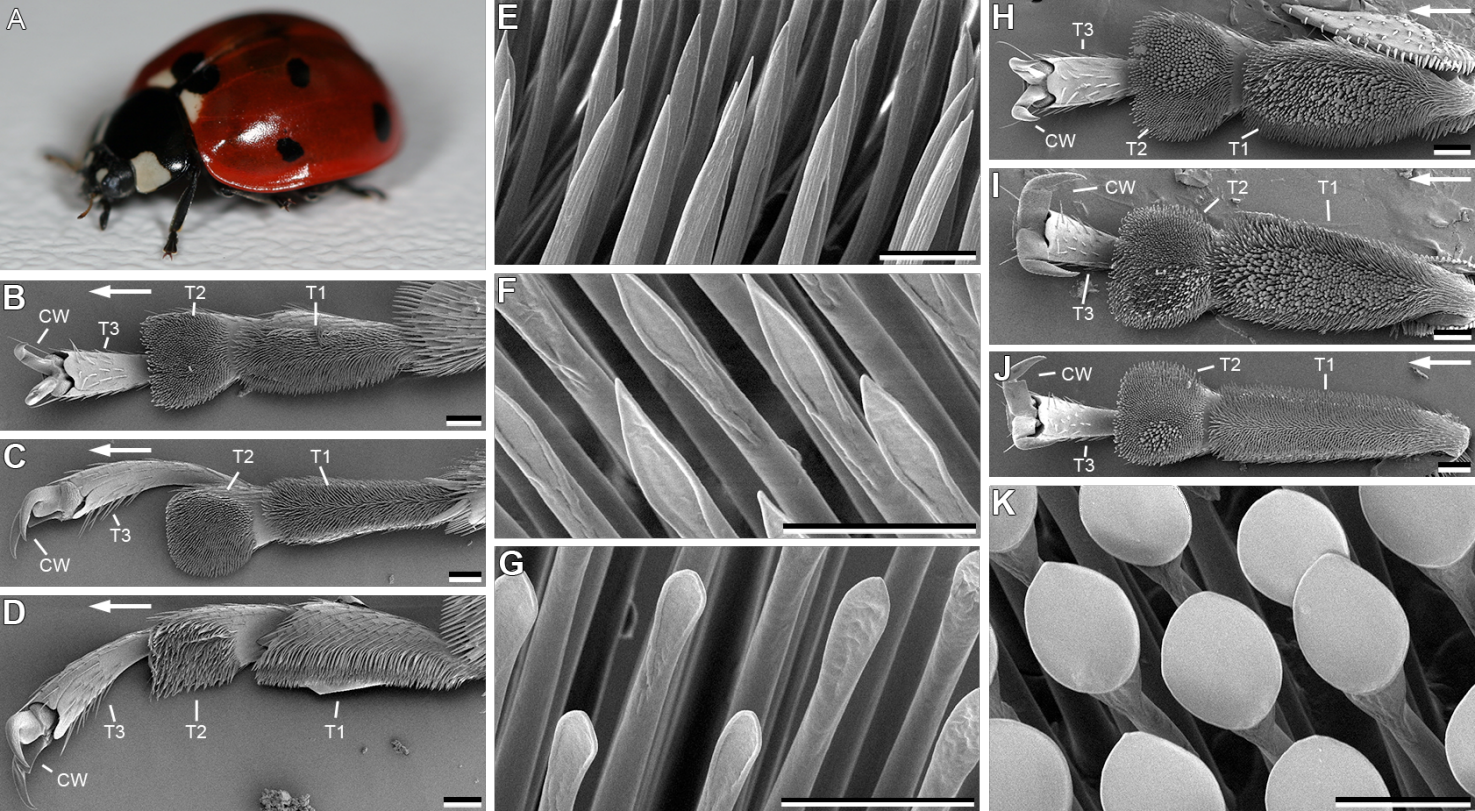
Attachment devices of *Coccinella septempuctata* (A) attachment devices. Tarsi of forelegs (B), midlegs (C), and hindlegs (D) in females are ventrally covered by different types of tenent setae (E–G). Tarsi of forelegs (H), midlegs (I), and hindlegs (J) in males. Tarsi of males were also ventrally covered by tenet setae types shown in (E, F), but have an additional type, which is terminated with discoidal terminal elements (K). CW, claws; T1, first proximal tarsomer; T2, second proximal tarsomer; T3, third proximal tarsomer. The arrows in (B–D) and (H–J) indicate distal direction. Scale bars in (B–D, H–J), 100 µm. Scale bars in (E–G, K), 10 µm.

**Figure 2:**
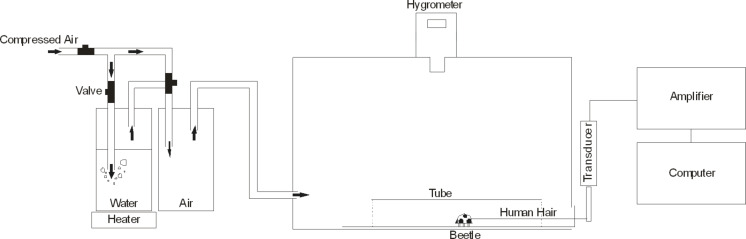
Schematic of the experimental setup used for traction force experiments under controlled ambient humidities. Tethered beetles were connected to a force sensor by a 15–20 cm long human hair. Humidity was controlled by mixing dry compressed air (approx. 15% relative humidity) and wet air. Wet air was produced by heating water in a glass bottle, in order to increase the amount of water vapour. Temperature was continuously monitored in the chamber to ensure that the use of the heater did not lead to temperature increase within the chamber. For details, see [14].

**Figure 3:**
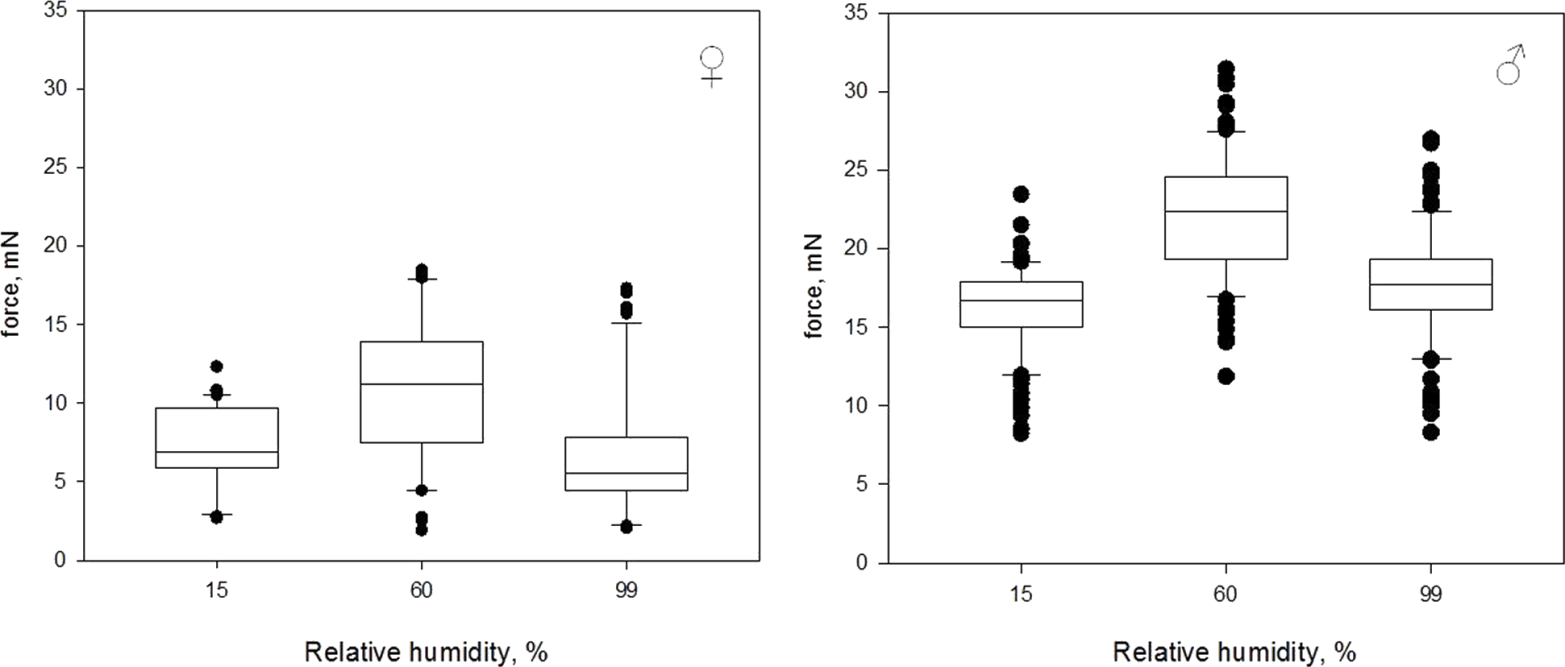
Box-and-whiskers plots, based on the results of the first experiment, with one RH level tested per day. The ends of the boxes deﬁne the 25th and 75th percentiles, with a line at the median and error bars deﬁning the 10th and 90th percentiles, black dots represent the outliers.

**Figure 4:**
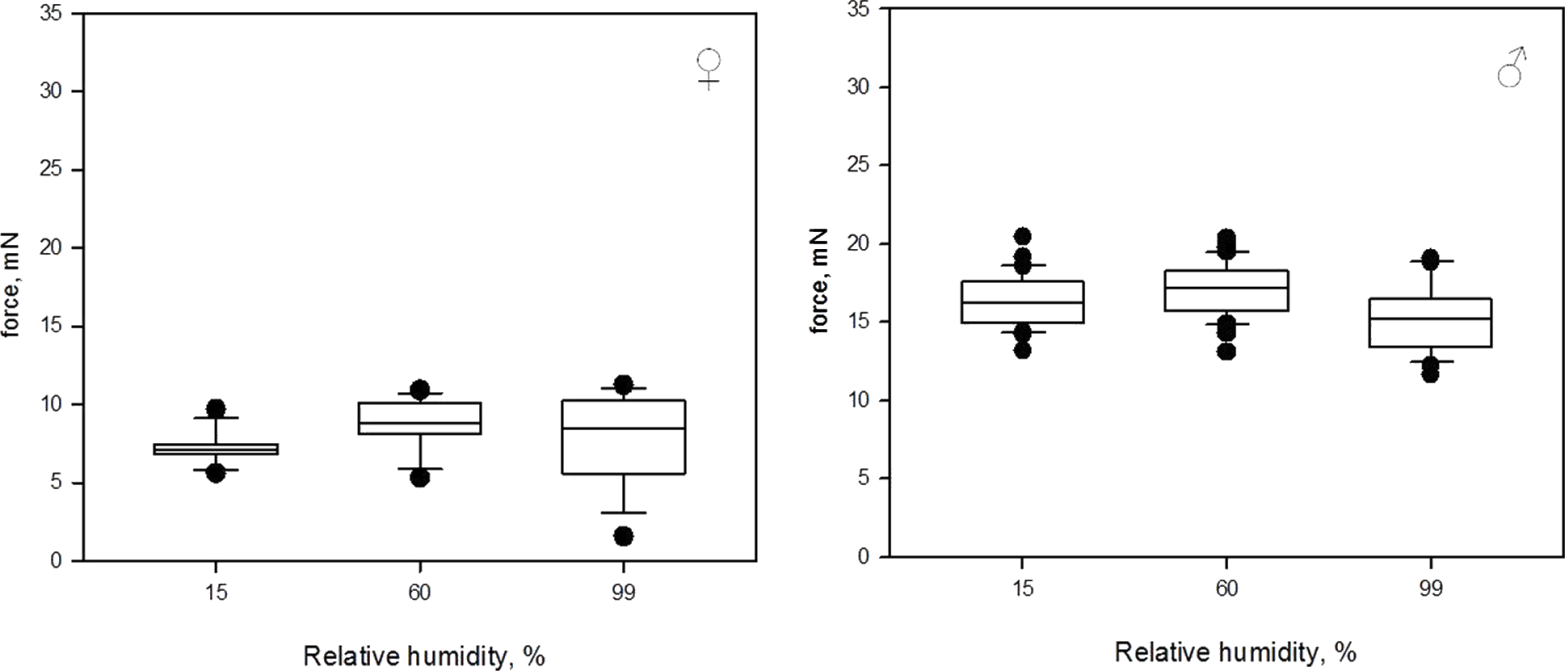
Box-and-whiskers diagrams based on the results of the second experiment with all three levels of RH tested at the same day. The ends of the boxes deﬁne the 25th and 75th percentiles, with a line at the median and error bars deﬁning the 10th and 90th percentiles, black dots represent the outliers.

For this study, the seven-spotted ladybird beetle *Coccinella septempunctata* LINNAEUS 1758 (Coleoptera, Coccinellidae) was chosen (Figure 1A). This beetle species is a generalist, living on diverse plant surfaces [38–39]. In their natural environment, beetles are exposed to various humidities, hence, their adhesive system must be well adapted to changing environmental conditions. Beetles were collected from bracken (*Pteridium aquilinum*) in the New Botanical Garden at Kiel University, Schleswig-Holstein, Germany. They were kept individually in plastic tubes at a temperature of 22–24 °C and relative humidity of 40–50%. These conditions were consistent with those at which the beetles were captured. The beetles were fed with honey. Figure 1 shows the hairy attachment devices of *C. septempunctata*. The tarsus is composed of three tarsomeres and two ventrally curved claws (Figure 1B–D,H–J). Only the first two tarsomeres (T1 and T2 in Figure 1) are ventrally covered by tenent setae. Different types of tarsal adhesive setae were distinguished: (1) setae with a pointed, filamentous tips (Figure 1E), (2) setae with lanceolate tips (Figure 1F), (3) setae with a flattened and rounded, spatula-shaped tips (Figure 1G), and (4) setae terminated with discoidal terminal elements (Figure 1K). All four types of tarsal adhesive setae were found in males. Females show the first three types only. Setae terminated with discoidal terminal elements were found in the centre of the first two tarsomeres (T1 and T2 in Figure 1) for all legs in males (Figure 1H–J), except for the first tarsomer (T1) of the hindleg (Figure 1J).

### Force measurements in a controlled atmosphere

Traction force experiments were performed in a polymethylmethacrylate (PMMA) chamber (30 × 14 × 14 cm) in which relative humidity could be manipulated by the controlled mixture of dry and wetted air (Figure 2, for details see [14]).

For this experiment, three levels of relative humidity (RH) were used: 15%, 60% and 99%. The RH was monitored with a P330 digital hygrometer (Dostmann electronic GmbH, Wertheim-Reicholzheim, Germany). To generate humid conditions, air was passed through a water bottle, so that the vapour concentration in the air increased. To reach 99% of relative humidity, slight heating was used to increase the amount of water vapour. This did not affect the temperature within the experimentation chamber. To generate a dry environment, dry air from a pressurized air pipe was pumped into the experimentation box. To stabilize ambient humidity and the thickness of adsorbed water films, the procedure was started about one hour before the experiments.

A strain-gauge force transducer (10 g capacity; World Precision Instruments, Inc., FL, USA) connected to a Biopac MP100 amplifier (Biopac System Inc., Goleta, CA, USA) was used to measure forces generated by the beetles. Force data were recorded with the AcqKnowledge 3.7.0 software (Biopac Systems Inc.). A half Plexiglas tube was installed inside the chamber, in order to guide beetle locomotion perpendicular to the force sensor and prevent it from climbing onto the lateral walls.

Prior to experiments the beetles were anesthetized with carbon dioxide and the elytra were glued together with a droplet of molten wax, to prevent them from flying (for details see [40]). Individual beetles were connected to the force transducer by a human hair (10–15 cm long) fixed to the elytra and to the force sensor with a droplet of molten wax. Beetles were placed on the Plexiglas bottom of the experimentation chamber and released such that they walk perpendicularly away from the force sensor. When the beetles were hold back by the strengthened hair they tried to pull forward, which led to transmission of traction forces to the force sensor. The traction force is dependent on the attachment ability of the pulling animal. This method has been used in previous studies [7,14,40]. We let the beetle pull for one minute and then repeated the experiment in a second run, to make sure that the performance of individual animals was stable. In total, 10 runs for each individual beetle for one minute at each of three humidity conditions were recorded.

The experiment was carried out in two different runs. The first run was done with 15 beetles (4 females and 11 males). All beetles were tested with one single level of humidity per day, further called Experiment 1. To exclude an influence of physiological conditions of individual animals on different experimental days, a second run was executed. In this run, 9 beetles (3 females and 6 males) were tested. Individual beetles were tested on one day at all three levels of relative humidity in a randomized fashion, further called Experiment 2. To evaluate the effect of the relative humidity on attachment force in both sexes of *C. septempunctata*, a Kruskal–Wallis One Way ANOVA on Ranks followed by an all pairwise multiple comparison procedure (Tukey Test, SigmaPlot 11.0, Systat Software Inc, San José, CA, USA) has been performed. In order to evaluate the effect of sex on the attachment force, a Mann–Whitney Rank Sum Test has been applied to the pooled data. Therefore, the data of the different relative humidity has been pooled individually for both experiments and sexes.

### Observations of the beetle behaviour at different relative humidities

A Plexiglas sheet was fixed with double side adhesive tape at three different positions: horizontal (0°), vertical (90°) and upside-down (180°) inside the experimentation chamber. We observed the locomotion behaviour of individual beetles on these surfaces. Additionally, to the three previously used humidity adjustments (RH of 15%, 60% and 99%), we made some observations at RH higher than 99%, when water began to condense on the substrate.

## Results

### Observational experiments

We observed that the beetle’s attachment is affected, as the water begins to condense on the substrate, when the relative humidity exceeds 99%. In this case, beetles were not able to hold on the vertical Plexiglas slide and slid along its surface.

#### Experiment 1: One level of relative humidity per day

The attachment ability of beetles was highest at an RH of 60% and lower for RH of 15% and 99% (Figure 3). Here and later data will be shown as median [minimum, maximum]. At an RH of 15%, females reached the median force of 6.9 mN [2.7 mN, 12.4 mN]. At an RH of 60%, the median force was 11.2 mN [1.9 mN, 18.6 mN]. At an RH of 99%, the median force was 5.5 mN [2.0 mN, 17.5 mN]. The forces measured at different RH differed significantly (Kruskal–Wallis One Way ANOVA on Ranks: *N* = 40; *H* = 30.035; *df* = 2; *P* ≤ 0.001). An all pairwise comparison (Tukey Test, see Table 1) revealed significant differences in measured traction forces between 15% RH and 60% RH as well as between 60% RH and 99% RH, but not between 15% RH and 99% RH.

**Table 1:** Statistics of the traction force measurements.

sex	humidity	Experiment	q-value	significance

female^a^	15% versus 60%	1	5.200	yes
female^a^	15% versus 99%	1	2.377	no
female^a^	60% versus 99%	1	7.577	yes
male^b^	15% versus 60%	1	15.634	yes
male^b^	15% versus 99%	1	3.776	yes
male^b^	60% versus 99%	1	11.858	yes

female^c^	15% versus 60%	2	6.003	yes
female^c^	15% versus 99%	2	3.473	yes
female^c^	60% versus 99%	2	2.530	no
male^d^	15% versus 60%	2	4.311	yes
male^d^	15% versus 99%	2	3.806	yes
male^d^	60% versus 99%	2	8.117	yes

female versus male^e^	pooled	1	*P* < 0.001	yes
female versus male^f^	pooled	2	*P* < 0.001	yes

^a–d^Kruskal–Wallis One Way ANOVA on Ranks with an all pairwise multiple comparison (Tukey Test). ^e,f^Mann–Whitney Rank Sum Test.

At an RH of 15%, males reached the median force of 16.7 mN [8.1 mN, 23.4 mN]. At an RH of 60%, the median force was 22.4 mN [11.7 mN, 31.4 mN]. At an RH of 99%, the median force was 17.7 mN [8.2 mN, 27.0 mN]. The forces measured at different RH differed significantly (Kruskal–Wallis One Way ANOVA on Ranks: *N* = 110; *H* = 133.1; *df* = 2; *P* ≤ 0.001). An all pairwise comparison (Tukey Test, see Table 1) revealed significant differences in measured traction forces for all levels of RH.

#### Experiment 2: Three levels of relative humidity per day

Similar to the first experiment, highest median attachment forces were observed at 60% RH (Figure 4). At an RH of 15%, females reached the median force of 7.1 mN [5.5 mN, 9.9 mN]. At an RH of 60%, the median force was 8.8 mN [5.3 mN, 11.0 mN]. At an RH of 99%, the median force was 8.5 mN [1.6 mN, 11.3 mN]. The forces measured at different RH differed significantly (Kruskal–Wallis One Way ANOVA on Ranks: *N* = 30, *H* = 18.167, *df* = 2, *P* ≤ 0.001). An all pairwise comparison (Tukey Test, see Table 1) revealed significant differences in measured traction forces between 15% RH and 60% RH as well as between 15% RH and 99% RH, but not between 60% RH and 99% RH.

At an RH of 15%, males reached the median force of 16.2 mN [13.2 mN, 20.4 mN]. At an RH of 60%, the median force was 17.2 mN [13.1 mN, 20.4 mN]. At an RH of 99%, the median force was 15.2 mN [11.6 mN, 19.1 mN]. The forces measured at different RH differed significantly (Kruskal–Wallis One Way ANOVA on Ranks: *N* = 60, *H* = 32.984, *df* = 2, *P* ≤ 0.001). An all pairwise comparison (Tukey Test, see Table 1) revealed significant differences in measured traction forces for all levels of RH.

### Effect of sex

A significant difference was detected between sexes (*P* < 0.001, Mann–Whitney Rank Sum Test, see Table 1), where males achieved two times higher forces than females at all three levels of RH. Thereby, the attachment ability of both males and females was likewise affected by ambient humidity.

## Discussion

The present study demonstrates, for the first time, that the performance of a wet (fluid supplemented) adhesive pad is influenced by ambient humidity. Our results indicate an optimal range of relative humidity with maximal traction forces in ladybird beetles. Similar observations have been made in dry adhesive pads of spiders, using a similar setup and method as in the present study [14], and geckos, revealed in measurements with living animals [11] and with isolated setae [10]. This is particularly interesting since both types of adhesive systems (wet and dry) are supposed to be based on different physical interactions (capillarity versus van der Waals forces).

For the dry adhesive pads of spiders and geckos the humidity-related effects on adhesion can be explained by three different mechanisms: (1) capillary forces due to the formation of liquid bridges; (2) changes in the effective short-range interactions due to adsorbed monolayers of water on the substrate [11,14]; (3) humidity-dependent material properties of insect cuticle and β-keratin (main constituent of gecko setae) [41–44]. In geckos, the effect of a RH on viscoelastic properties of the setal shaft was shown [13]. It was argued that with an increasing humidity the viscoelastic bulk energy dissipation increases within setae while being pulled off the substrate, leading to higher resistance of the adhesive contact [12,45].

For *C. septempunctata* it was recently shown that the setal tips contain high amounts of the soft rubber-like protein resilin [46], which is a hygroscopic protein capable of binding high amounts of water [47]. Peisker et al. [46] showed a 6000-fold increase in the Young’s modulus of the setal tips after drying. Consequently, the degree of hydration should also significantly affect the mechanical properties of the setae of the beetles in the present study and may also explain our results on ladybird beetles, at least the increase in traction forces from 15% RH to 60% RH.

Two other observations may further support the hypothesis that a change in material properties with increasing humidity is responsible for at least the increase in traction forces from 15% RH to 60% RH. Voigt et al. [48] observed significantly higher forces generated by beetles (*Leptinotarsa decemlineata*) on a dry surface, which were kept prior the experiment under humid conditions if compared to beetles kept prior the experiment under dry conditions. In the present study we observed a distinct difference between both types of experiments (Experiment 1 and 2). The differences between the measured traction forces, although significant, are smaller in case of all humidity levels measured on one day (Experiment 2), than in the case of one humidity level measured per day (Experiment 1). Since in Experiment 2 the humidity levels were randomized for individual runs, there were some beetles that were measured first at higher humidity and then at lower humidity. In combination with relatively short durations between individual runs, those individuals, according to the idea of changing material properties, should then show a better performance also at the lower humidity level if compared to Experiment 1, thus, reducing the observable differences between different humidity levels.

Although the hypothesis of changing material properties seems to be quite plausible, there are also several aspects speaking against it. The tarsal secretions in hairy attachment pads of beetles were found to be mainly lipid-based [22,26–27,49–50], with only a small volatile fraction (likely water) [30]. Peisker et al. [46] speculated that these secretions may have an additional function, apart from forming capillary bridges, which is to cover setal tips and thereby preventing the desiccation of setal tips, thus, keeping them in a soft rubber-like state. In this light, it seems unlikely that the setal tips absorb water from ambient humidity. However, this hypothesis has not been confirmed so far and little is known about where tarsal liquids are actually secreted and delivered in hairy attachment pads of beetles. Moreover, assuming capillarity to be responsible for the generated traction forces, it is not clear how the proposed increase in the viscoelastic bulk energy dissipation with increasing humidity should affect the attachment ability in this case, since the contact is mediated by the tarsal secretion and not by a solid–solid contact.

Coming back to the abovementioned mechanisms of capillary forces and/or changes in the effective short-range van der Waals interaction, which might influence the humidity dependent adhesion in geckos [10], it needs to be mentioned that both mechanisms require water to be present at the contact interface. It can be either absorbed water on the substrate, whose actual amount depends on the relative humidity and on the surface chemistry [19,51] or capillary condensation [52]. Due to the high content of lipids in the tarsal secretions of the beetles, a direct effect of water capillary bridges can be neglected. Though, it cannot totally be excluded that the composition and properties of the tarsal secretion are altered by ambient humidity. Thus, for example, different contributions from viscous forces could be expected [31,53]. For *C. septempunctata*, viscosity of the tarsal secretion was found to be of order 20 mPa·s [31] and droplets of volumes around 1 fL of the tarsal secretion made contact angles smaller than 10° with freshly cleaved mica surfaces (calculation based on droplet geometries reported in [30]). Moreover, it is known that micro-emulsions may have complex structures depending on the particular water-to-oil ratio [54], which might also have significant effects on beetle adhesion. Indeed, it has been shown that the adhesive pad fluids of insects contain minute droplets of water that are crucial for the function of the adhesive system [28].

The reduction in traction forces observed in the present study at 99% RH (without visible water condensation) may be explained by a sufficient amount of adsorbed water on the substrate. It has been shown that the friction behaviour between two solids crucially depends on the water layer thickness between them [55]. At critical thickness of adsorbed monolayers water becomes liquid-like, which may render the onset of an “aquaplaning effect”. Accordingly, in adhesion experiments of setal pads, adhesion rapidly dropped as soon as water condensation became visible on the substrate or moistening of substrate and/or pad surface ([14,56] and this study).

In nature, animals are constantly exposed to certain humidity. Most of insects live on plants containing a humid boundary layer in the vicinity of the plant surface. The humidity close to leaf surfaces ranges between 30% and 75% [57]. It is probable that the insects’ adhesive pad is adapted to work most efficient under these conditions.

Finally, comparing absolute values in the attachment forces of *C. septempunctata* beetles, we found evidence for a sexual dimorphism. Male beetles generated higher adhesion than females, which was previously reported and explained by the presence of specialized setae with discoidal tips that can generate higher adhesion on smooth substrates due to their advantageous, geometry-induced homogenous stress distribution [58–60]. Similar results were previously obtained for chysomelid beetles [5,61]. The dimorphism is explained by the need of males to stay attached to the smooth elytrae of females during copulation and mate guarding. However, the attachment ability of males and females is similarly affected by humidity and, thus, underlying mechanisms of humidity dependent adhesion seem to be similar in both sexes.

## Conclusion

The present study demonstrates, for the first time, the effect of ambient humidity on a wet adhesive pad that is supplemented by a lipid-based secretion. This shows that humidity similarly affects the function of both dry and wet adhesive pads. However, the underlying mechanisms leading to this effect remain unclear. To be able to distinguish between different mechanisms (changing material properties, water layer thickness, changing tarsal secretion properties), experiments with substrates of different surface free energy should be performed in the future.
